# Capsid redirection mechanism of the *Staphylococcus aureus* pathogenicity island SaPIpT1028

**DOI:** 10.1098/rstb.2024.0075

**Published:** 2025-09-04

**Authors:** Adaeze Doris Ojiogu, Jonasz B. Patkowski, Xu Kuang, Tiago R. D. Costa, Jakob T. Rostøl, José R. Penadés

**Affiliations:** ^1^ Imperial College London, London SW7 2AZ, UK

**Keywords:** SaPI, phage, gene transfer

## Abstract

*Staphylococcus aureus* pathogenicity islands (SaPIs) are prototypical members of the phage-inducible chromosomal islands (PICI) family. These elements redirect helper phage capsid assembly to produce smaller capsids, accommodating the satellite genome while excluding the phage genome. This study identifies how SaPIpT1028 mediates capsid redirection through a unique gene, *rcm* (redirecting capsid morphogenesis). While *rcm* has no sequence similarity to known capsid assembly regulators, our results demonstrate that its expression is necessary and sufficient for redirecting capsid morphogenesis in *S. aureus* phages, such as φ7206. We show that, to do this, Rcm interacts with the φ7206 major capsid protein. Comparative evolutionary and structural analyses reveal functional parallels between Rcm and CpmB, a regulator used by other SaPIs. However, Rcm has evolved a multi-helical topology to match the multi-helical topology of the scaffold protein of φ7206. Sequence homology and AlphaFold predictions suggest that Rcm competitively interacts with the φ7206 scaffold protein, altering capsid size through a mechanism akin to CpmB. This work highlights SaPI adaptation, exemplified by Rcm’s ability to exploit phages resistant to other remodellers, while inhibiting their reproduction. These findings underscore the dynamic co-evolution of phages and SaPIs, with Rcm playing a pivotal role in capsid size regulation and phage interference.

This article is part of the discussion meeting issue ‘The ecology and evolution of bacterial immune systems’.

## 1. Introduction

Phage satellites are mobile genetic elements that depend on a helper virus for their replication and lifecycle. Examples include *Escherichia coli* P4 [1,2], the widely distributed family of phage-inducible chromosomal islands (PICIs) [3,4] and *Vibrio* satellites, such as PICI-like elements (PLEs) [5] and phage-inducible chromosomal minimalist islands (PICMIs) [6]. Typically about one-third the size of their associated phages, satellites have evolved various strategies to ensure their transmission, often at the expense of the helper phages [7]. This dynamic significantly impacts both phage and bacterial evolution and ecology [8]. In *Staphylococcus aureus*, these elements can also carry and disseminate a broad range of virulence and resistance genes, such as superantigens, host adaptation factors and antimicrobial resistance determinants, potentially transforming non-pathogenic bacterial strains into pathogenic ones [9]. In an evolutionary context, satellites engage in highly efficient DNA transfer mechanisms, such as lateral transduction and cotransduction [10], which enhance chromosomal mobility. Ecologically, they contain immune systems that regulate horizontal gene transfer by either facilitating or restricting it [11,12], and they also influence the biology of other mobile genetic elements, including phages and plasmids [8,13,14].

In most of the satellites studied, satellite DNA is packaged within capsids made from phage-encoded proteins. This creates competition between satellites and helper phages for the same protein resources. To enhance their own propagation, satellites disrupt the reproductive cycle of the helper phage in various ways. A key tactic used by these elements to facilitate their transfer, while hindering that of their helper phage, is the production of smaller capsids. These capsids are of a size that allows only the satellite genomes to be fully packaged, excluding the much larger helper phage genomes. For instance, P4 achieves this through the expression of Sid [15], while PLEs rely on the production of TcaP [16]. In *S. aureus*, PICIs (known as SaPIs in this species) express either CpmAB [17–19] (for *pac* phages) or Ccm [20,21] (for *cos* phages), depending on the packaging system used by their helper phages. PICIs present in other species, such as *Enterococcus faecalis* [22] or *Pasteurella multocida* [3], also produce satellite-sized small capsids, but the genes or mechanisms involved in this process remain unknown.

Since the formation of small-sized capsids appears to be a common feature of many satellites, and given that even within PICIs present in the same species, multiple strategies for phage capsid redirection have been discovered (e.g. CpmAB or Ccm in SaPIs), we initiated this study to identify additional mechanisms of capsid modulation in *S. aureus*. We were particularly interested in the mechanism used by SaPIpT1028 to promote its transfer. This island piqued our curiosity for several reasons: first, it is the prototypical member of PICIs encoding immune systems whose expression regulates horizontal gene transfer [11]; second, SaPIpT1028 does not encode the previously characterized cpmAB or ccm capsid redirection factors. Finally, a previous study revealed that the classical *S. aureus* phage 80α can induce and package this island, although packaging occurs in large capsids [11]. We hypothesized that this island might be unable to redirect 80α capsid formation to produce small capsids, but it could potentially do so with other phages encoding different packaging proteins. This turned out to be the case. In this study, we identify the novel SaPI-encoded protein Rcm (redirecting packaging morphogenesis) (previously ORF7) involved in redirecting phage capsid assembly to produce SaPI-specific small capsids. Importantly, although different in sequence, our results indicate that this novel protein functions through a conserved mechanism similar to other satellite-encoded proteins that promote the formation of small-sized capsids. Overall, our findings represent a striking example of convergent evolution, and highlight the complex mechanisms by which phage satellites manipulate their host phages.

## 2. Results

### (a) SaPIpT1028 ORF7 interferes with helper phage reproduction by reprogramming capsid formation

**Figure 1.**
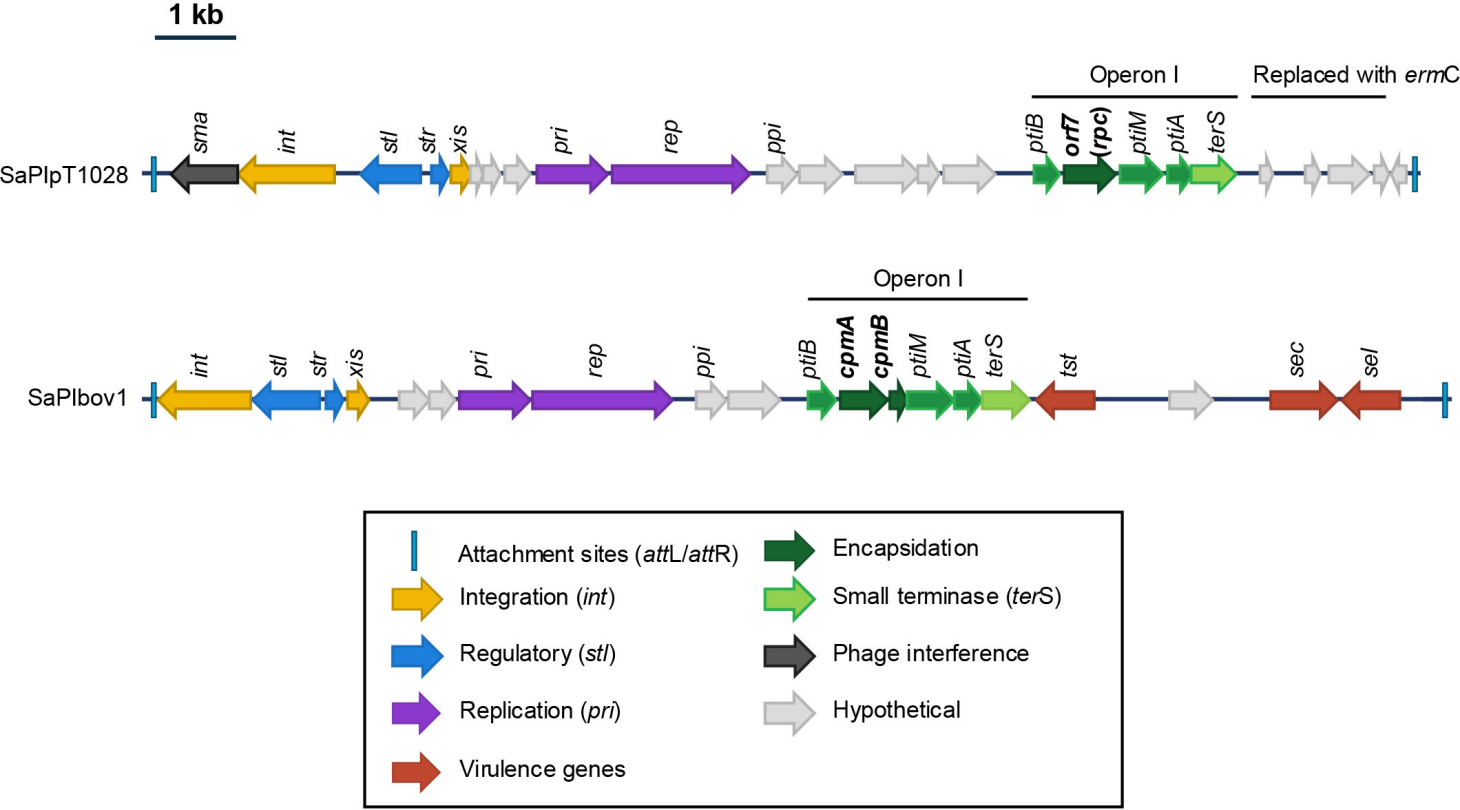
Genetic maps of SaPIpT1028 and SaPI1. According to the prophage convention, genomes are aligned with the integrase gene (*int*) at the left end. Genes are coloured according to their sequence and function: *int* and *xis* are yellow; transcription regulators are blue; replication genes (including the primase gene (*pri*) and the replication initiator (*rep*) are purple; genes affecting expression (*pti*) or assembly (*cpm*) of helper phage virion components are dark green and light green respectively; the terminase small subunit gene (*terS*) is in mid-green; genes encoding hypothetical proteins are grey. The genes encoded in the respective operon I are highlighted.

SaPIs that use the headful mechanism for packaging typically contain what is known as operon I, which usually encodes six different genes [17]. These genes contribute to the formation of SaPI-sized capsids (*cpmA* and *cpmB*) [17], interfere with phage reproduction (*ptiA*, *ptiB* and *ptiM*) [23], or promote SaPI packaging (*terS*; figure 1) [24]. In the SaPIpT1028-encoded operon I, which is almost identical to that found in the prototypical SaPIbov1 (figure 1), we observed that instead of the *cpmA* and c*pmB* genes responsible for small-sized capsid production, SaPIpT1028 carried *orf7* in the same location. The protein encoded by *orf7* shows no similarity to any of the previously characterized SaPI proteins involved in helper phage capsid redirection.

**Figure 2.**
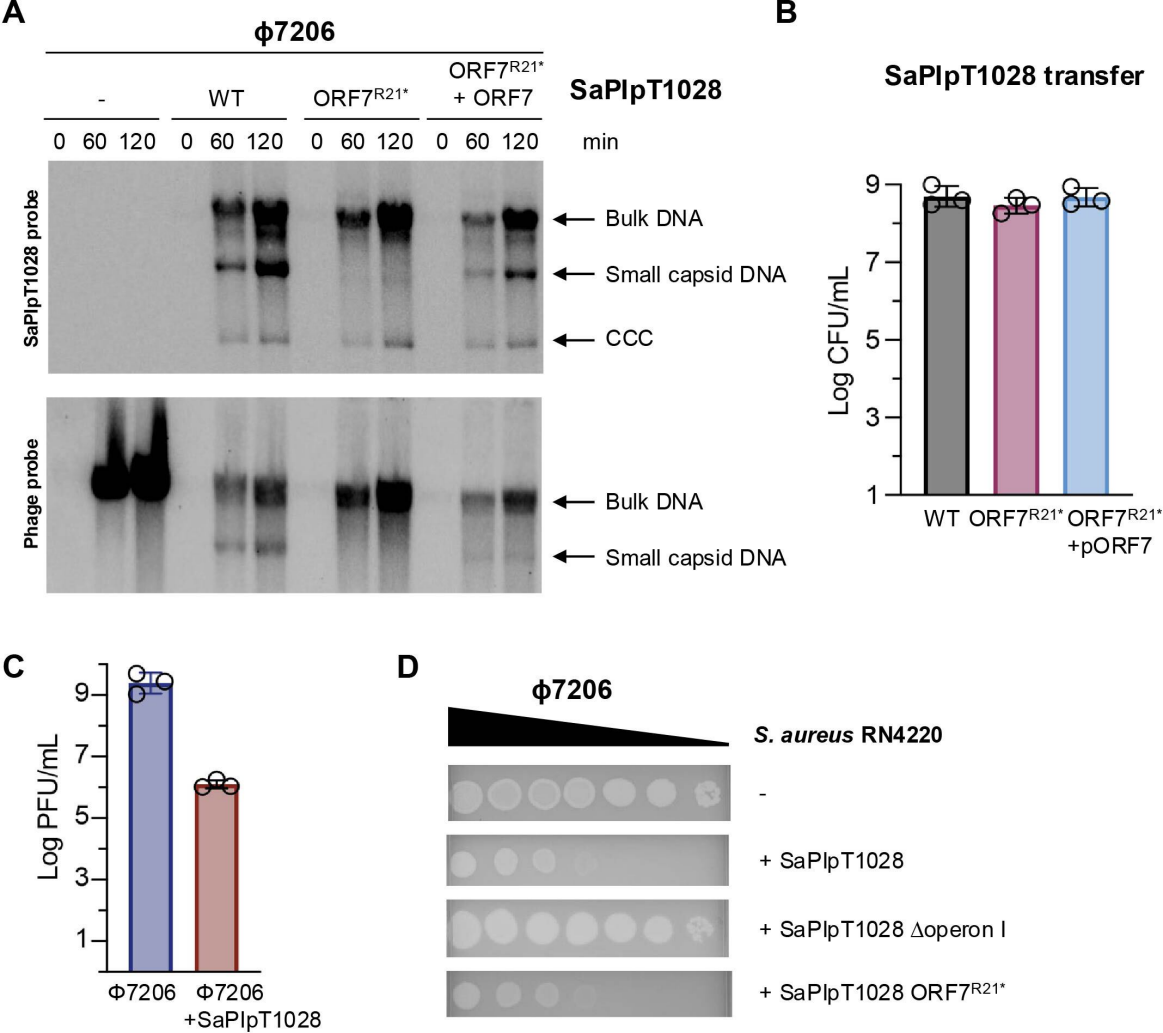
SaPIpT1028 ORF7 (Rcm) interferes with helper phage infection by promoting small capsid formation. (A) *Staphylococcus aureus* lysogenic strains for φ7206, carrying different versions of SaPIpT1028 (wild-type (WT), mutant in ORF7 (R21*), or mutant in ORF7 complemented with ORF7), were induced with mitomycin C. Bacterial cells were taken at the indicated time points (min), and processed to extract their DNA, which was then separated on a 0.7% agarose gel and Southern blotted using probes specific for the SaPIpT1028 (upper panel) or φ7206 (lower panel) genomes. Bulk DNA represents the concatemeric form. Small capsids correspond to DNA packaged into SaPI-sized capsids. CCC represents covalently closed circular molecules. (B) SaPIpT1028 transfer. SaPIpT1028 titres following induction of φ7206 in the presence of the indicated SaPIpT1028 versions. Bars represent the mean of three biological replicates, error bars represent s.d. (C) φ7206 prophage induction in the absence or presence of SaPIpT1028, as quantified on RN4220. Bars represent the mean of three biological replicates; error bars represent s.d. PFU, plaque-forming units. (D) Representative spot assay for φ7206 phage onto recipient bacteria carrying the indicated SaPIpT1028 variants. Tenfold serial dilutions of phage lysates were applied.

We hypothesized that if the SaPIpT1028 ORF7 had a similar role to CpmAB in redirecting capsid assembly, it would be possible to obtain a helper phage for SaPIpT1028, which, after induction and in the presence of the island, would generate small-sized SaPI particles. To test this, we constructed an RN4220 strain harbouring a φ7206 (accession code: PP098624) prophage and SaPIpT1028, and a control strain with only the prophage. Mitomycin C (MC) induction of φ7206 in the presence of SaPIpT1028, but not in its absence, resulted in specific small-sized SaPIpT1028 or phage DNA bands when analysed by Southern blotting (figure 2A), suggesting the generation of small-sized capsids that primarily (but not exclusively) contained SaPIpT1028 DNA. This characteristic is not unique to SaPIpT1028; it has been previously observed during the packaging of the classical *pac* islands SaPI1 and SaPIbov1 [25,26]. As expected, since φ7206 induced SaPIpT1028, transfer of the island occurred at high frequencies (figure 2B), which was associated with a significant reduction in the reproduction of the helper phage after induction (figure 2C). This interference was confirmed in experiments when a φ7206 lysate was used to infect RN4220 derivative strains with or without SaPIpT1028. As shown in figure 2D, the presence of the island significantly reduced the ability of φ7206 to infect the recipient cells, demonstrating that SaPIpT1028 interferes with the φ7206 life cycle.

To confirm that the observed phenotype was due to one of the genes encoded in the SaPIpT1028 operon, we repeated the previous experiments using a SaPIpT1028 derivative mutant in *orf7* (*orf7*^R21*^). As a control, the strain carrying the SaPIpT1028 *orf7*^R21*^ derivative was complemented ectopically with a plasmid expressing *orf7*. Mutation of *orf7* eliminated the production of small-sized particles, while complementation of the mutant strain restored its ability to generate SaPI capsids (figure 2A). Importantly, mutation of *orf7* did not reduce the transfer of SaPIpT1028 (figure 2B), as it is expected that several copies of the SaPI genome can be packaged in the larger phage-sized capsids as concatemers [25,26].

Moreover, while φ7206 was able to reproduce normally in a mutant strain where all the genes in operon I were deleted, deletion of *orf7* alone did not allow the helper phage to replicate in the presence of SaPIpT1028 *orf7*^R21*^ (figure 2C), suggesting that the island employs multiple operon I-encoded mechanisms to interfere with this phage.

### (b) ORF7 is necessary and sufficient to redirect phage capsid assembly

**Figure 3.**
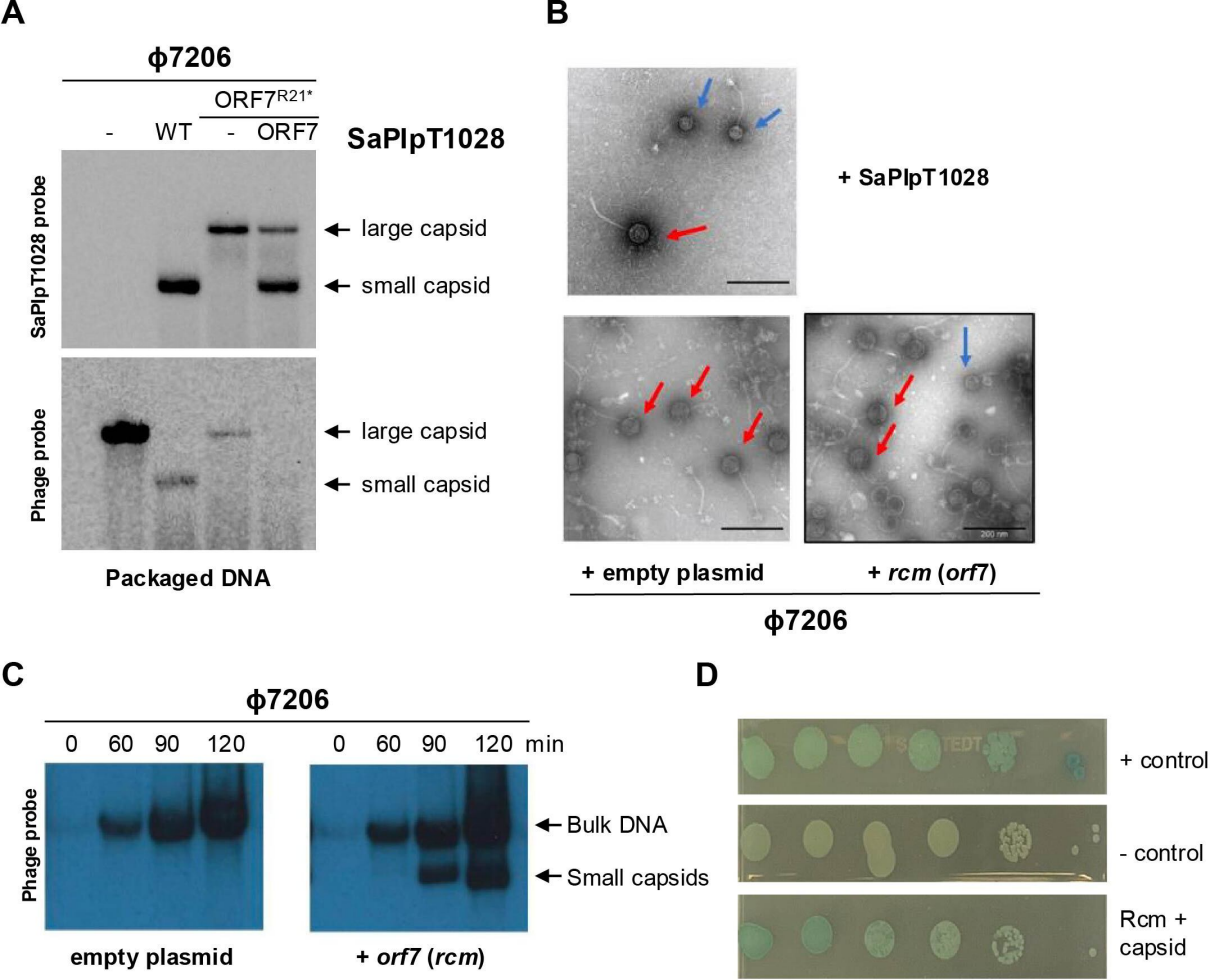
Rcm is necessary and sufficient for the production of the SaPI-sized capsids. (A) Lysogenic strains for φ7206, either alone or carrying different versions of SaPIpT1028 (WT, ORF7^R21*^, or ORF7^R21*^ complemented with ORF7), were induced with MC. The lysates obtained after induction were precipitated, and the packaged DNA was extracted, separated on a 0.7% agarose gel and subjected to Southern blotting using probes specific for the SaPIpT1028 (upper panel) or φ7206 (lower panel) genomes. (B) Electron microscopy analysis of the mitomycin C-induced lysogen strains for φ7206 carrying SaPIpT1028, empty plasmid or a plasmid expressing *rcm*. Phage- and SaPI-sized particles are highlighted with red and blue arrows, respectively. (C) *Staphylococcus aureus* strains lysogenic for φ7206, carrying empty vector or a vector expressing *orf7* (*rcm*), were induced with mitomycin C. Bacterial cells were taken at the indicated time points (min), and processed to extract their DNA, which was then separated on a 0.7% agarose gel and Southern blotted using a probe specific for the φ7206 genome. Bulk DNA represents the concatemeric form. Small capsids correspond to DNA packaged into SaPI-sized capsids. (D) The *rcm* and phage capsid genes were cloned into pUT18c and pKNT25, respectively. Plasmids were co-transformed into *Escherichia coli* strain BTH101. Serial dilutions of an overnight culture were plated onto lysogeny broth (LB) supplemented with kanamycin, ampicillin, 100 μM isopropyl β-d-1-thiogalactopyranoside (IPTG) and 20 μg ml^−1^ 5-bromo-4-chloro-3-indolyl-β-d-galactopyranoside (X-gal). BTH101 transformed with pUT18c-zip and pKNT25-zip or pUT18c and pKNT25 served as positive or negative controls for protein–protein interactions, respectively.

Next, to confirm the presence of small-sized SaPI capsids in the lysates obtained in the presence of SaPIpT1028, we first precipitated the packaged DNA from lysates obtained after induction of strains either lysogenic for φ7206 only, or carrying the wild-type (WT) or *orf7* mutant versions of SaPIpT1028. The obtained DNA was analysed by Southern blot using DNA probes specific for the SaPIpT1028 or φ7206 DNAs. While in the absence of SaPIpT1028 only a high-molecular-weight band containing phage DNA was observed, in the presence of SaPIpT1028, the most prevalent band corresponded to the small-sized SaPI capsids, which, confirming previous results, mainly (but not exclusively) contained SaPI DNA (figure 3A). This experiment also clearly demonstrated the severe interference the island exerted on the phage, as the phage-sized DNA band almost disappeared in the presence of the WT island. The analysis of the SaPIpT1028 *orf7* mutant and complemented strains confirmed the role of ORF7 in the production of the small-sized capsids (figure 3A).

To visualize the capsid formation of SaPIpT1028 and its helper phage, we used electron microscopy (EM) to analyse particles resulting from the MC induction of a bacterial culture lysogenic for φ7206 carrying SaPIpT1028. As predicted, we observed the presence of small-sized SaPI particles (figure 3B, top panel), demonstrating how SaPIpT1028 leads to the formation of smaller capsids. Small capsids were also observed when ORF7 was expressed from a plasmid, confirming that ORF7 is sufficient to redirect packaging.

The previous results indicated that ORF7 was necessary for the formation of the SaPI-sized capsids. However, they did not rule out the possibility that other proteins encoded by SaPIpT1028 were also involved in this process. To test whether ORF7 was necessary and sufficient, we either introduced the plasmid expressing ORF7 or an empty plasmid into the φ7206 lysogen. After MC induction of the prophage and expression of the cloned gene, we analysed the presence of SaPI particles by EM (figure 3B, bottom panels), which showed that ORF7 alone could redirect capsid formation. To corroborate this, we induced the same strains, harvested the lysate upon cell lysis and isolated the packaged DNA from capsids for analysis by Southern blot. Importantly, in the presence of ORF7, a band of small size appeared, corresponding to the size of the small SaPI capsid (figure 3C). Together, these experiments confirm that the expression of ORF7 is sufficient to redirect φ7206 capsid assembly. Therefore, we decided to rename *orf7* as *rcm*, for redirecting capsid morphogenesis.

### (c) SaPIpT1028 Rcm interacts with the φ7206 major capsid protein

Since previously characterized satellite-encoded proteins involved in the production of the small-sized capsids interacted with the phage-encoded major capsid protein [15,18], we hypothesized that a similar scenario would occur with Rcm. To test whether Rcm interacts with the major capsid protein of φ7206 (GenBank accession number QFJ68405; 274 aa), a bacterial adenylate cyclase-based two-hybrid (BACTH) assay was performed, in which *rcm* was cloned as bait in plasmid T18, while the phage-encoded proteins were cloned as prey in plasmid T25. As shown in figure 3D, the co-expression of ORF7 and the major capsid φ7206 proteins resulted in blue colonies, indicating an interaction between the two proteins and supporting the hypothesis that ORF7 binds to the major capsid φ7206 protein.

### (d) Structural characterization of Rcm and its interaction with the major capsid protein of φ7206

**Figure 4.**
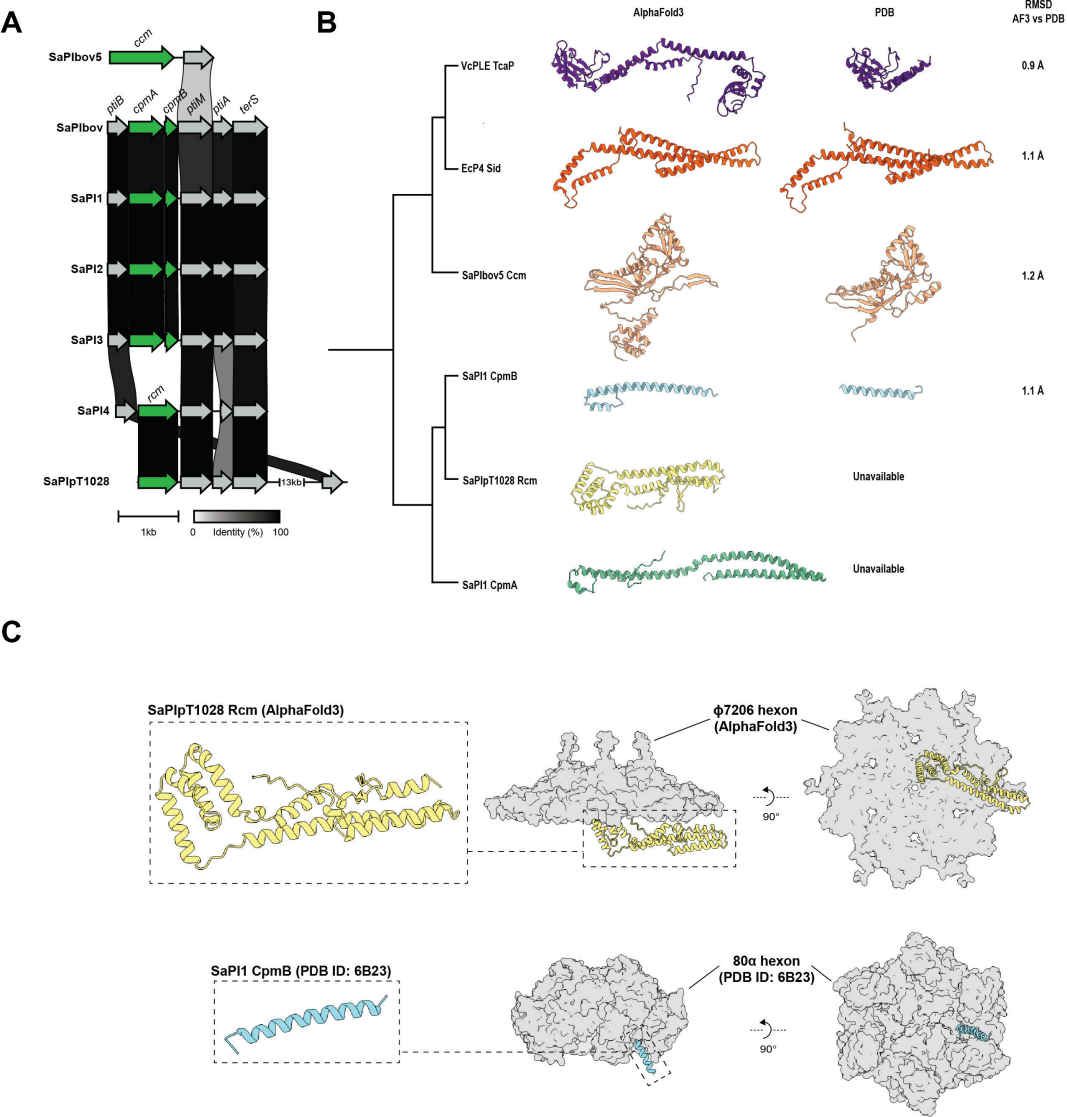
Evolutionary origin of Rcm, its structural prediction and comparison with CpmB. (A) Alignment of the operon I or operon I-like loci among different SaPI, including SaPIbov5 (GenBank: HM228919), SaPIbov (GenBank: AF217235), SaPI1 (GenBank: U93688), SaPI2 (GenBank: ED010993), SaPI3 (GenBank: AF410775), SaPI4 (NCBI: NC_002952) and SaPIpT1028 (GenBank: AY954948), showing a consistent localization of a capsid size switcher protein (green arrows) immediately before *ptiM*, including *rcm* in SaPIpT1028. (B) Amino acid sequence homology tree accompanied by AlphaFold 3 prediction models of known capsid size symmetry switchers, including CpmA and CpmB from SaPI1, Ccm from SaPIbov5, Sid from *Escherichia coli* satellite phage P4, TcaP from phage-inducible chromosomal island-like element (PLE) of *Vibrio cholerae,* together with Rcm of SaPIpT1028, showing homology of the latter to specifically CpmB. (C) Top: AlphaFold 3 prediction model of Rcm from SaPIpT1028 without (left) and with (right) the prediction model of φ7206 phage capsid hexon. Bottom: structure of CpmB from SaPI1 (PDB ID: 6B23) without (left) and with (right) the structure of 80α procapsid hexon. Similarly to CpmB, Rcm shows a predominantly alpha-helical structure and is predicted to localize to the inner face of the hexon.

To better understand the evolutionary origins of Rcm, we performed homology alignments against other SaPI genomes and their respective capsid size switchers. The organization of operon I, the segment of SaPI genome responsible for phage interference, varies among different SaPIs, yet consistently positions the protein responsible for capsid size switching immediately before *ptiM* (figure 4A). This includes the previously reported Ccm from SaPIbov5 [20] and CpmAB from SaPI1-3 [18], as well as the novel capsid switcher Rcm from SaPIpT1028, which we show to interfere with the capsid of phage φ7206. When differences between switchers (Ccm, CpmAB and Rcm) are ignored, SaPIpT1028 operon I shows near 100% sequence identity across all components with SaPIbov and SaPI1-3, which all use the CpmAB capsid size switcher, while it is clearly more distantly related to SaPIbov5, which instead uses the Ccm switcher (figure 4A). This suggests a closer evolutionary relationship between the interference mechanism of CpmAB- and Rcm-dependent satellites than those employing Ccm, hinting that Rcm might have evolved as a close alternative to CpmAB—an adaptation that enables interference with CpmAB-insensitive phages, such as φ7206. Interestingly, during the analysis, an identical Rcm was found in SaPI4, suggesting its capacity to interfere with phage φ7206.

To better understand the similarities between the different switchers, we employed amino acid sequence homology using ClustalOmega and UniProt Align (figure 4B) together with the accompanying amino acid sequence identity matrix (electronic supplementary material, figure S1a). The tree was complemented by AlphaFold 3 prediction models of the known capsid symmetry switchers, including TcaP from phage-inducible chromosomal island-like element (PLE) of *Vibrio cholerae* [16], Sid from *E. coli* satellite phage P4 [15], SaPIbov5-encoded Ccm [20,21] and SaPI1-encoded CpmAB [18], along with SaPIT1028 Rcm (figure 4B). The structural predictions exhibited high to very high confidence values (predicted local distance difference test (pLDDT) < 70) for most proteins except TcaP (electronic supplementary material, figure S1b), with the prediction of Rcm assuming the highest predicted template modelling score (pTM) value of 0.8 (electronic supplementary material, figure S1b). The predictions are accompanied by all the known deposited structures of the switchers, showing nearly identical atomic models when compared with their respective predictions (figure 4B). Notably, the experimentally obtained models are devoid of some unresolved or truncated stretches; hence models of TcaP, Ccm or CpmB are shorter compared with the respective predictions (figure 4B).

Rcm and CpmB cluster together in the tree and exhibit the highest relative percentage identity (at 28.57%) compared with the rest of the switchers (figure 4B and electronic supplementary material, figure S1a). This might suggest a shared functional mechanism between Rcm and CpmB. The Rcm prediction model exhibits multi-alpha-helical topology compared with the also alpha-helical albeit much smaller topology of CpmB (figure 4B). Amino acid alignment between Rcm and CpmB shows some shared motifs, especially motifs [YELN] and [FGL] towards the C-terminus of Rcm (electronic supplementary material, figure S2a), while structural alignment reveals root mean square deviation (RMSD) of 0.97 Å across best aligning (pruned) 36 atom pairs and 16.37 Å across all 72 pairs (electronic supplementary material, figure S2b). Further AlphaFold 3 multimer predictions of the φ7206 capsid with Rcm suggest a potential interaction site beneath the hexon, with segments of Rcm protruding outside in a manner similar to CpmB (figure 4C) (PDB ID: 6B23) [18]. While confidence of that prediction is low (pTM = 0.44, interface predicted template modelling score (ipTM) = 0.4) (electronic supplementary material, figure S3), this positioning is significant, as CpmB’s protrusion beyond the hexon enables homodimerization, directly contributing to phage capsid size alteration. We therefore hypothesize that Rcm could undergo a similar dimerization event; however, structural data are needed to test this hypothesis.

**Figure 5.**
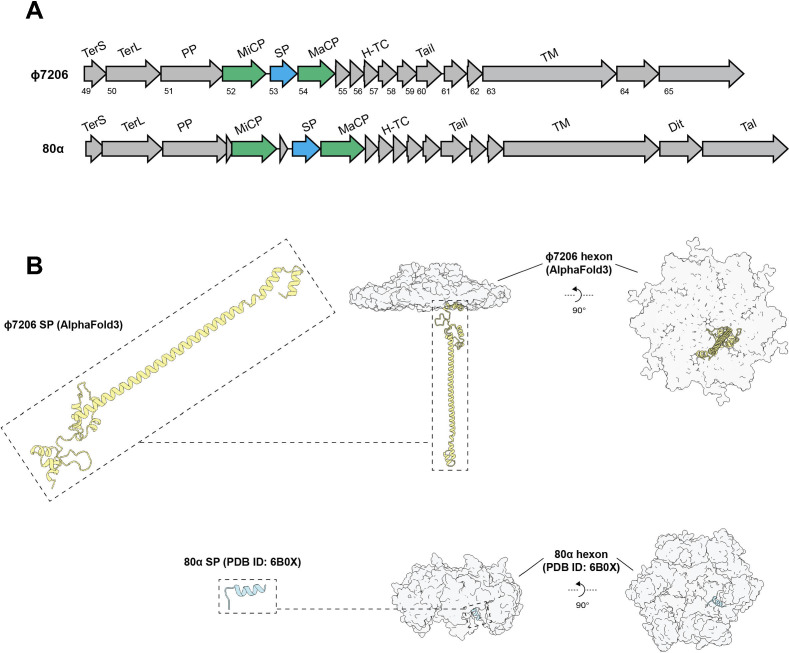
Identification of a putative scaffold protein of phage φ7206 and comparison with that of 80α. (A) Segments of φ7206 (top) and 80α (bottom) phage genomes, showing a similar organization of genes encoding proteins such as small and large terminases (TerS and TerL), portal proteins (PP), minor and major capsid proteins (MiCP and MaCP), scaffold proteins (SP), head–tail connectors (H-TC), tail subunits and tape measure (TM). A putative SP was identified within the φ7206 operon (blue arrow), sandwiched between the MiCP and MaCP (green arrows) by analogous localization in the experimentally tested SP of phage 80α. (B) Top: structural prediction of φ7206 SP by AlphaFold 3 without (left) and with (right) the φ7206 hexon prediction. Bottom: structure of 80α SP (PDB ID: 6B0X) without (left) and with (right) the 80α hexon structure.

Together, the sequence homology and structural resemblance to CpmB imply a comparable functional mechanism of Rcm. The primary recruitment mechanism of CpmB into the 80α phage capsid is its competitive advantage over the native scaffold protein (SP), attributed to the high homology and conserved motifs shared between them, facilitating replacement [18]. Based on our hypothesis that Rcm operates in a manner akin to CpmB, we searched for an SP in the φ7206 operon that would exhibit similarities with Rcm. A putative SP was identified in φ7206, positioned between the minor and major capsid proteins, a location identical to that of phage 80α (figure 5A). Structurally, this putative SP presents a larger, multi-helical topology when compared with the smaller SP of phage 80α (figure 5B), which might be the reason why φ7206 remains insensitive to CpmAB interference and only upon evolving a much larger switcher, such as Rcm, it is possible to interfere with φ7206. Further supporting our hypothesis of a CpmB-like mechanism of Rcm is the sequence alignment between φ7206 SP and Rcm (electronic supplementary material, figure S4a), which reveals conserved motifs distributed along both proteins, resembling the relationship between SaPI1 CpmB and the cognate 80α SP, which can engage in competition because of the conserved [RIIK] motif (electronic supplementary material, figure S4b) [18].

Collectively, the similarity between Rcm and CpmB, and the existence of the putative φ7206 SP with homology to Rcm support our hypothesis of a CpmB-like mechanism of SaPIpT1028 Rcm interference with phage φ7206 capsid assembly. In contrast to CpmB, Rcm exhibits a much larger structure, which might have evolved as a way for SaPIpT1028 to bypass the φ7206 insensitivity to CpmB. In this scenario, possessing a multi-helical SP, such as that of φ7206, provides resistance to smaller switchers, such as CpmB, therefore, requiring a larger and multi-helical switcher, such as Rcm. We postulate that similar mechanisms might be observed across other phage–SaPI interactions, whereby the topology of the switcher will reflect the topology of the cognate SP, maintaining the CpmB-like mechanism of switcher versus SP competition.

## 3. Discussion

With the exception of a recently discovered family of PICIs, the capsid-forming PICIs (cf-PICIs), which encode all the proteins required for the formation of their small-sized capsids and the packaging of cf-PICI DNA into these capsids [27], all previously described phage satellites rely on phage-encoded proteins for packaging. With the exception of PICMIs [6], satellites typically encode one or two proteins that redirect the assembly of phage-encoded proteins to produce small-sized capsids. These capsids allow the packaging of the smaller satellite genomes, but not the larger phage genomes. Importantly, as demonstrated here and in prior studies [17,21], the production of small-sized capsids does not enhance the transfer of PICIs, helping to explain why some satellites do not produce small capsids. However, this process represents a crucial mechanism of phage interference [28], central in the role satellites play in interfering with the life cycle of their helper phage.

In response to such interference, it can be anticipated that phages encoding capsid proteins capable of evading this interference would be positively selected. Subsequently, PICIs may evolve to encode proteins capable of interacting with the new capsid proteins, driving an evolutionary arms race between helper phages and their satellites. This co-evolutionary dynamic could account for the existence of multiple variants of satellite-encoded proteins that redirect phage capsid assembly. Thus, it is tempting to speculate that the mechanisms of capsid redirection identified thus far represent only the tip of the iceberg and that many novel mechanisms remain to be discovered.

While producing small-sized capsids does not appear to directly impact satellite transfer, it has a significant effect on plasmid evolution [14]. Previous studies have demonstrated that non-conjugative plasmids exhibit a bimodal size distribution, i.e. the size of plasmids is similar either to PICI genomes or to phage genomes [29]. This allows the plasmids to hijack these particles for their dissemination to other cells through generalized transduction, the main mode of mobility for non-conjugative plasmids. Thus, in the presence of active satellites, phage-sized plasmids lose their ability to spread owing to their inability to be packaged in the redirected small capsids, while PICI-sized plasmids can still spread. In turn, such larger plasmids, which often encode AMR genes and virulence factors, will be less mobile, with implications for bacterial evolution.

The broader role of satellites as active participants in microbial genome plasticity, which exert pressures that affect not only their phage hosts [8] but also other mobile genetic elements [14], highlights their important ecological significance. It is not surprising that satellites have therefore evolved highly effective mechanisms of capsid redirection through competitive interference. The relatively simple and structurally minimal alpha-helical protein CpmB encoded by SaPI1 and SaPIbov mimics the structural topology of the helper phage 80α’s SP—a key component of its capsid assembly. This mimicry allows CpmB to outcompete the native SP, redirecting 80α’s standard capsid assembly pathway to produce smaller capsids [18], which in turn ensures the preferential packaging of the smaller satellite genome while physically excluding the larger 80α phage genome. At the molecular level, this mechanism relies on the structural compatibility between the satellite-encoded symmetry switcher, such as CpmB, and the phage-encoded SP, emphasizing the importance of such compatibility in facilitating efficient interference. From the perspective of the phage, however, the reduction of its transmission owing to satellite interference will pose positive selective pressure to develop capsids that are resistant to symmetry switchers. Will the satellites respond?

Indeed, here we document a novel symmetry switcher encoded by SaPIpT1028, the protein Rcm, which shares evolutionary ancestry with CpmB. Rcm has undergone significant structural diversification adapted to manipulate the assembly machinery of the φ7206 phage, which encodes a larger and multi-helical SP. This exemplifies the ongoing co-evolution between satellites and their helper phages, where structural adaptations in capsid switchers must mirror the multi-helical topology of their targeted SP by evolving more convoluted topologies that evolved likely as attempts to minimize interference. From the satellite perspective, the growing specificity between its switcher and the cognate SP not only ensures efficient redirection but also reduces competition from other co-residing mobile genetic elements that may rely on the same helper phage. This reflects an adaptation to minimize resource overlap, allowing Rcm to exert precise control over the φ7206 capsid assembly process and maintaining exclusivity. This, however, imposes an important trade-off as highly specialized satellites undergo a host range reduction, explaining why SaPIpT1028 cannot interfere with phage 80α, unlike the closely related SaPIbov or SaPI1. Furthermore, the observed attempts of the phage at developing resistance to satellites introduce an exciting venue for additional phage-encoded defence systems, for example aimed at blocking the activity of the switcher, particularly considering the size of the phage genome is significantly less constricted than that of the satellite. Further structural and biochemical studies will be essential to elucidate the exact mechanism of Rcm, shedding light on the diverse strategies satellites employ to manipulate phage capsid assembly.

With the discovery of Rcm, the anti-phage arsenal of SaPIpT1028 is further expanded [11]. Like most satellites, SaPIpT1028 interferes with the life cycle of its helper phage, reducing the phage titre and serving as a *bona fide* bacterial immune mechanism. As described in this report, Rcm plays a central role in this process. Previously, SaPIpT1028 was also shown to carry a defence system, Sma (single-protein MazF-like antiphage system), which is expressed when the SaPI is lysogenic and protects against non-helper phages [11]. Rcm and Sma thus protect against different threats (helper and non-helper phages, respectively), and offer the host cell and the SaPI multiple layers of protection.

However, as noted before, Rcm is not active against certain helper phages, such as 80α, where the helper phage capsid protein is incompatible with Rcm. In this case, and in the absence of other interference mechanisms, both the helper phage and SaPIpT1028 would be mobilized to high levels. This can be beneficial to both entities, as they are more likely to ‘travel together’ and infect the same naive cells, co-existing in the recipient chromosome. In this way, the host cell, the phage and the SaPI can all benefit from their cumulative protection from external mobile genetic elements [11]. Thus, by encoding Rcm, which only strongly blocks some helper phages, SaPIpT1028 can diversify its potential lifestyle options, ensuring survival and propagation under different conditions.

## 4. Material and methods

### (a) Bacterial strains and growth conditions

The bacterial strains, plasmids and oligonucleotides used in this study are listed in electronic supplementary material, tables S1–S3, respectively. *Staphylococcus aureus* strains were grown at 37 or 30°C on tryptic soy agar (TSA) or in tryptic soy broth (TSB) with shaking (120 r.p.m.), supplemented with erythromycin (10 µg ml^−1^, Sigma-Aldrich) or chloramphenicol (10 µg ml^−1^, Sigma-Aldrich) as needed. *Escherichia coli* strains were grown at 37°C on lysogeny broth (LB) agar, supplemented with ampicillin (100 µg ml^−1^, Sigma-Aldrich) and kanamycin (30 µg ml^−1^, Sigma-Aldrich) when required.

### (b) Molecular methods

All DNA manipulations followed standard molecular biology protocols. The oligonucleotides used in this study are listed in electronic supplementary material, table S3 and were obtained from Sigma-Aldrich. Restriction enzymes and T4 DNA ligase were purchased from New England Biolabs, and DNA sequencing was performed by Eurofins.

### (c) Plasmid construction

Plasmid construction followed established methodologies. Desired inserts were amplified using oligonucleotides listed in electronic supplementary material, table S3, purified with the QIAquick PCR Purification Kit (Qiagen) and cloned into their respective plasmids.

The SaPIpT1028 *orf7* (*rcm*) gene was amplified from SaPIpT1028 and cloned into the expression plasmid pCN51 under the control of a cadmium-inducible promoter (Pcad), generating pJP2908. All plasmid constructs were confirmed through Sanger sequencing (Eurofins).

### (d) DNA manipulations

Gene deletions in *S. aureus* were performed as previously described [30,31]. In-frame deletion mutants were generated using the allelic exchange vector pBT2-βgal and the oligonucleotides listed in electronic supplementary material, table S3.

For SaPIpT1028, plasmid pJP2902, carrying flanking regions of the target genes in the SaPIpT1028-like operon I, was electroporated into *S. aureus* RN4220 carrying an SaPIpT1028 derivative marked with an erythromycin resistance cassette (JP19047).

A point mutation (ochre mutation) for SaPIpT1028 *orf7* (*rcm*) was introduced by inserting a stop codon into the open reading frame of the *rcm* gene to generate a non-functional Rcm protein. For identification, the mutation was watermarked with silent restriction sites downstream of the stop codons. The plasmid was electroporated into *S. aureus* RN4220 (JP19047). Transformants were selected on TSA plates containing chloramphenicol and erythromycin, incubated at 32°C to maintain temperature-sensitive plasmids, and subjected to homologous recombination at 42°C. Resulting mutants were confirmed by PCR and DNA sequencing.

### (e) Phage plaque assays

*Staphylococcus aureus* strains carrying pCN51 or pJP2908, as well as various SaPIpT1028 mutants, namely JP21517 (∆operon I-like) and JP21518 (Orf7^R21*^), were cultured overnight in TSB supplemented with erythromycin (10 µg ml^−1^) for the selection of SaPIpT1028. For plasmid-containing strains, 1 µM CdCl₂ was added to induce transcription from the pCN51 promoter. An overnight culture was diluted 1 : 50 in fresh TSB and grown to an OD_540_ of 0.34. Bacterial lawns were prepared by mixing 300 µl of cells with phage top agar (PTA) and spreading the mixture onto square plates. Serial dilutions of phages were prepared in phage buffer (50 mM Tris pH 8, 1 mM MgSO₄, 4 mM CaCl₂, 100 mM NaCl) and spotted onto the plates, which were incubated at 37°C for 24 h.

### (f) Phage and SaPI induction

*Staphylococcus aureus* lysogenic strains carrying SaPIpT1028 (JP19690), SaPIpT1028 *orf7*^R21*^ (JP21624) or the mutant complemented *in trans* with an inducible plasmid carrying *orf7* (JP21691) were cultured overnight in TSB. A 1 : 50 dilution of the overnight culture was prepared in fresh TSB and grown to an OD_540_ of 0.15−0.2. Mitomycin C (MC) (2 µg ml^−1^) was added to induce φ7206. For plasmid-containing strains, CdCl₂ (1 µM) was added to activate *orf7* expression. After 6 h of induction, lysates were filtered using 0.2 µm filters (Sartorius Stedim Biotech) to obtain lysates containing phage and SaPI particles.

### (g) SaPI transduction

To quantify SaPI particles, recipient *S. aureus* RN4220 cells (OD_540_ ≈ 1.4) were infected with serially diluted lysates in phage buffer, incubated at 37°C for 20 min, mixed with TSA top agar (TTA; 6 g TSB and 1.5 g agar; Oxoid; 200 ml) and poured onto TSA plates supplemented with erythromycin and 17 mM sodium citrate. For plasmid samples, CdCl₂ (1 µM) was added. Colonies were counted to determine the number of transduction particles in the lysate, expressed as colony-forming units per millilitre (c.f.u. ml^−1^).

### (h) Southern blot analyses

Induced samples were collected, lysed and subjected to DNA extraction and agarose gel electrophoresis. DNA was depurinated, denatured and transferred to a nylon membrane (Hybond-N, Amersham). Detection probes for SaPIpT1028 or phage DNA were generated by PCR using oligonucleotides listed in electronic supplementary material, table S3. Membranes were hybridized with digoxigenin (DIG)-labelled probes and visualized using the CSPD substrate (Roche).

### (i) Electron microscopy

Electron microscopy was performed to assess the role of the island carrying a mutation in *orf7* in capsid morphogenesis of φ7206. φ7206 lysogens carrying SaPIpT1028 (JP19690), an empty plasmid (JP21865) or a plasmid expressing ORF7 (JP21864) were cultured overnight. A 1 : 50 dilution of the overnight culture was prepared in fresh TSB, grown to an OD_540_ of 0.15−0.2, and φ7206 was induced with MC and CdCl₂ for ORF7 expression. Induced cultures were incubated at 30°C with gentle shaking for 4 h or overnight at room temperature until completely lysed. After filter sterilization to remove unlysed cells, 30 ml of lysates were treated with DNase (1 µg ml^−1^) and RNase (1 µg ml^−1^) for 30 min at room temperature. Lysates were polyethylene glycol-precipitated (PEG 8000/500 ml lysate) and subjected to caesium chloride density-gradient centrifugation, as previously described [27]. The purified material was negatively stained with 1% uranyl acetate and examined using an FEI Tecnai F20 electron microscope operated at 200 kV with a typical magnification of 65 500×. Images were captured on a Gatan Ultrascan 4000 CCD camera at the School of Life Sciences, MVLS, University of Glasgow. Photos were obtained at 200 nm.

### (j) Bacterial adenylate cyclase two-hybrid (BACTH) assay

The interaction between Rcm and the φ7206 major capsid protein was assessed using the BACTH system. Constructs fusing these proteins to adenylate cyclase fragments (T18/T25) were co-transformed into *E. coli* BTH101 cells grown on LB plates containing ampicillin (100 µg ml^−1^), kanamycin (30 µg ml^−1^), 5-bromo-4-chloro-3-indolyl-β-d-galactopyranoside (X-gal) (40 µg ml^−1^) and isopropyl β-d-1-thiogalactopyranoside (IPTG) (500 µM) to induce full expression of the hybrid proteins. Plates were incubated at 32°C for 2 days. A single colony from these plates was used to inoculate 5 ml of LB broth supplemented with the same antibiotics and incubated at 32°C for 18−24 h. Tenfold serial dilutions of the overnight culture were prepared in LB broth, and 10 µl of each dilution was spotted onto LB plates containing ampicillin, kanamycin, X-gal and IPTG. Plates were incubated at 32°C for 2 days. Blue colonies indicated a positive interaction.

### (k) Bioinformatical analysis

Genome alignments were performed using clinker [32], while protein alignments were performed in ClustalOmega phylogeny tree [33] and visualized in FigTree (http://tree.bio.ed.ac.uk/software/figtree/). Protein predictions were done in AlphaFold 3 [34] and visualized in UCSF ChimeraX [35].

## Data Availability

Materials and data are provided as electronic supplementary material available online [36].
